# The Influence of Neurotrophins on the Brain–Lung Axis: Conception, Pregnancy, and Neonatal Period

**DOI:** 10.3390/cimb46030160

**Published:** 2024-03-15

**Authors:** Federica D’Amico, Cecilia Lugarà, Giovanni Luppino, Carlo Giuffrida, Ylenia Giorgianni, Eleonora Maria Patanè, Sara Manti, Antonella Gambadauro, Mariarosaria La Rocca, Tiziana Abbate

**Affiliations:** Pediatric Unit, Department of Human Pathology in Adult and Developmental Age “Gaetano Barresi”, AOUP G. Martino, University of Messina, Via Consolare Valeria 1, 98124 Messina, Italy; federicadamico93@gmail.com (F.D.); cecilialug@gmail.com (C.L.); giovilup97@gmail.com (G.L.); carlo.giuffrida@studenti.unime.it (C.G.); ylenia.g18@gmail.com (Y.G.); elepat1995@gmail.com (E.M.P.); gambadauroa92@gmail.com (A.G.); mariarosarialarocca93@gmail.com (M.L.R.); tiziana.abbate93@gmail.com (T.A.)

**Keywords:** neurotrophins, NTs, brain–lung axis, airway, neurodevelopment, BPD, SIDS, CCHS

## Abstract

Neurotrophins (NTs) are four small proteins produced by both neuronal and non-neuronal cells; they include nerve growth factor (NGF), brain-derived neurotrophic factor (BDNF), neurotrophin-3 (NT-3), and neurotrophin-4 (NT-4). NTs can exert their action through both genomic and non-genomic mechanisms by interacting with specific receptors. Initial studies on NTs have identified them only as functional molecules of the nervous system. However, recent research have shown that some tissues and organs (such as the lungs, skin, and skeletal and smooth muscle) as well as some structural cells can secrete and respond to NTs. In addition, NTs perform several roles in normal and pathological conditions at different anatomical sites, in both fetal and postnatal life. During pregnancy, NTs are produced by the mother, placenta, and fetus. They play a pivotal role in the pre-implantation process and in placental and embryonic development; they are also involved in the development of the brain and respiratory system. In the postnatal period, it appears that NTs are associated with some diseases, such as sudden infant death syndrome (SIDS), asthma, congenital central hypoventilation syndrome (CCHS), and bronchopulmonary dysplasia (BPD).

## 1. Introduction

Neurotrophins (NTs) are small proteins formed of dimers of about 13,500 Da [1]. These molecules include nerve growth factor (NGF), brain-derived neurotrophic factor (BDNF), neurotrophin-3 (NT-3), and neurotrophin-4 (NT-4) [2]. NTs have a common structure characterized by a pair of antiparallel B-strands that generate an elongated shape stabilized by three disulfide bridges. Such structures are composed of highly conserved cystine pairs, called cystine knots [3,4]. Despite the similarities among NTs, important structural differences exist due to ionized acidic or basic residues exposed on the surface. Some of these molecular differences affect the interaction with different receptors as well as their pharmacokinetic behavior [5].

Over the last few decades, many authors have studied the importance of NTs in the connection between the brain and the respiratory system, emphasizing their impact in both physiological and pathological development processes.

In our previous work [5] we focused our attention on the influence of NTs over respiratory and neurological processes. In this review, we aimed to summarize the most remarkable studies on the role of NTs in the development of the brain–lung axis, especially during pregnancy and the neonatal period. We combined the terms “neurotrophins” OR “NTs” AND “brain- lung axis” OR “airway” OR “neurodevelopment” in a computerized search of PubMed limited to the last 20 years, with the aim of providing an overview of the existing knowledge, based on a critical evaluation without standardized methodologies or statistical analyses.

## 2. Synthesis, Maturation, and Secretion of NTs

NTs are produced by both neuronal and non-neuronal cells as preproproteins and cleaved into pro-NTs by intracellular proprotein convertases (metalloproteinases). This process begins in the rough endoplasmic reticulum, where pro-NTs (constituting 210 to 270 amino acids) are packed into secretory vesicles [3,6]. Subsequently, they are processed by metalloproteinases to mature NTs, consisting of 120 amino acids [3,7]. However, in some cases, the precursors may be cleaved at the extracellular level [3,8,9].

## 3. NT Receptors

NTs operate through two types of mechanisms: a genomic one, consisting of a slow pathway regulating cell growth and survival, and typically inducing nuclear transcription processes and gene expression, and a non-genomic mechanism, which is faster and occurs in the cytoplasm as a consequence of the activation of rapid signaling [5,8]. NTs interact with four receptors (Table 1): p75 neurotrophin receptor (p75NTR), Tropomyosin receptor kinase A (TrkA), Tropomyosin receptor kinase B (TrkB), and Tropomyosin receptor kinase C (TrkC) [5,10,11].

P75NTR is a 75 kDa receptor belonging to the tumor necrosis factor (TNF) receptor superfamily; it binds all NTs and pro-NTs with low affinity. When NTs bind p75NTR, a cascade of events is activated, leading to cell apoptosis.

Trk receptors (140 kDa) are members of the neurotrophic tyrosine kinase (NTRK) receptor family and bind the mature forms of NTs with higher affinity, and are regulated by p75NTR [3,5,12]. NTs binding to Trk receptors results in the autophosphorylation of tyrosine residues, leading to the activation of signaling pathways such as RAS/MAP/ERK, phosphoinositide-3 kinase (PI3K)/Akt (protein kinase B), phospholipase C phosphoinositide-3 kinase/Akt (protein kinase B), and phospholipase C Gamma (PLCy). These signaling pathways have a crucial role in cell differentiation, apoptosis, and cell replication. Trk receptors exert their action through genomic mechanisms [5,13,14]. The three Trk receptors interact differently with NTs: NGF preferentially binds TrkA; NT-3 binds and activates TrkC with higher affinity; BDNF and NT-4 preferentially bind TrkB [3,5].

Conversely, non-genomic mechanisms act faster and depend principally on NTs binding to high-affinity Trk receptors. Moreover, NTs can directly modulate ion channels, membrane receptors, and other fundamental mechanisms of neuronal replication, differentiation, and apoptosis [5,13,14].

NT receptors are expressed on neurons and glial cells in both the central and peripheral nervous systems. These receptors are also expressed in non-neural tissues, and can be found in the skin, smooth and striated muscle, respiratory airway, gastrointestinal system, and urogenital system [15].

## 4. NTs: Expression and Function

NTs are classically considered to be functional molecules specific to the nervous system [16,17]. However, recent research showed that they are expressed in different structures, such as the skin, skeletal and smooth muscle, respiratory airway, as well as some structural cells. In addition, NTs are involved in several physiological and pathological conditions concerning different anatomical sites [15,18].

### 4.1. NTs and the Brain

Over the last few decades, numerous studies have demonstrated NTs’ crucial role in both the neonatal and adult brain. Several functions of the mature nervous system are regulated by the interaction between NTs and different neurotransmitter systems. In addition, NTs are involved in different neurological diseases, during both the developmental stage and adulthood, due to NTs’ role in neurogenesis, neuroplasticity, synaptic function, and neuronal differentiation and survival [5].

During pregnancy, the three major actors (mother, placenta, and fetus) are responsible in different ways for the production of NTs. In the fetus, NTs are involved in the development of the brain and respiratory system [19]. In the third month of pregnancy (9th–10th weeks) specific glutamatergic neurons start expressing p75NTR in the cortical plate, the region containing neurons that will give rise to cortical layers 2 to 6 [20]. Studies on animal models have shown that NT receptors are expressed in the early stages of neuronal development: during this period, NT production in target tissues seems to regulate neuronal survival or apoptosis [21]. BDNF is copiously expressed in the central nervous system (CNS), where it influences neuronal growth and morphology. This NT is involved in neural circuit development and in synaptic and structural plasticity [22]. In the fourth month of pregnancy, BDNF promotes neuronal growth in the frontal lobe of the CNS and, in combination with NT-3, regulates vessel maintenance and angiogenesis.

NTs, in particular, BDNF and NT-4, play a key role in activity-dependent neuronal synaptic plasticity [23]. Long-term potentiation (LTP), defined as a long-lasting activity-dependent enhancement in synaptic strength, is involved in learning and memory processes [24]. In the cortical neurons, LTP induction results in increased levels of BDNF and its TrkB receptor’s mRNA due to repetitive stimulation patterns able to increase intracellular calcium [25]. This interaction between NTs and their Tkr receptors occurs in the hippocampus, which plays a central role in memory acquisition. Intracellular signaling pathways are triggered by BDNF and NT-3 in the presynaptic and postsynaptic neurons. The link between NTS and Tkr encourages both the mitogen-activated protein kinase (MAPK) pathway and the activation of phospholipase C gamma. These pathways culminate in the activation of transcription factors, resulting in the synthesis of new proteins and the promotion of actin polymerization, crucial in inducing structural modifications to the synapses [26]. In the presynaptic membrane, BDNF increases the arrival of synaptic vesicles and modulates the SNARE complex, the protein complex that regulates the fusion of synaptic vesicles permitting the release of neurotransmitters. In the postsynaptic membrane, NTs enhance AMPA and NMDA glutamate receptors, which are crucial in the LTP mechanisms [27,28].

In the hippocampus, BDNF/Trk activation could be a potential mechanism of epileptogenesis promotion. BDNF promotes the sprouting of the GABAergic mossy fiber of dentate granule cells, increasing seizure susceptibility. In adulthood, it was suggested that a decrease in BDNF levels may be related to the development of neurodegenerative disorders such as Alzheimer’s, Parkinson’s, and Huntington’s diseases [28,29,30]. In childhood, a reduction in BDNF/Trk activity seems to be implicated in the development of autism spectrum disorders (ASD) and intellectual disability (ID) [31,32]. In these neurological diseases, it is possible to find an increase in NT-4 levels in neonatal blood. NT-4 is crucial for neuronal survival, proliferation, and differentiation, and for synaptic plasticity. It also promotes the survival of motor and sensory neurons, the outgrowth of neurite ganglia in the retina, and synapse formation in the hippocampus [33]. NGF, the first described and best characterized NT, induces the activation of the Raf pathway by interacting with TrkA receptor. It also has a crucial role in neurite outgrowth and development, and in the survival of cholinergic neurons in the CNS as well as the nociceptive peripheral neurons [34].

### 4.2. NTs and the Lungs

In recent years, research has shown increasing interest in studying NTs’ and their receptors’ roles in different physiological and pathological pulmonary conditions. NTs and their receptors are expressed in the lungs at different concentrations and with different functions according to the specific lung cells and/or structures (nerves, immune cells, bronchial epithelium, airway smooth muscle (ASM), fibroblasts, and vascular endothelium) (Table 2) [35].

The airway epithelium produces NTs primarily under the action of inflammatory cytokines. In fact, the airway epithelium may be considered a major source of NGF and BDNF, as demonstrated by their elevated levels in the lungs of mice following repeated allergen challenges and in bronchial biopsies of asthmatics [36]. NTs’ receptors (especially Trk receptors) are less expressed in the airway epithelium, while they are more expressed in nerves innervating the airway smooth muscle and pulmonary neuroendocrine cells. Particularly, immunocytochemical studies confirmed the expression of NGF, BDNF, NT-3, and their specific receptors, primarily TrkA. These data suggest that the NTs produced in the airway epithelium act preferentially in the adjacent tissues, such as smooth muscle, inflammatory, and nerve cells. Furthermore, some studies have demonstrated that inflammatory processes increase NT synthesis by the airway epithelium, while corticosteroids, which have anti-inflammatory action and are also used in asthma treatment, limit this production [35,37,38]. NTs also play an important role in the regulation of epithelial cell proliferation and survival. In fact, NGF promotes Clara cell proliferation and enhances the repair process in the lungs [38], while the absence of NGF seems to increase epithelial cell injury and death in cases of respiratory syncytial virus (RSV) infection [39]. In addition, BDNF and NT-3 stimulate human bronchial epithelial cells to produce nitric oxide (NO), which is involved in the bronchodilation mechanism. The latter process is promoted by both non-genomic and genomic mechanisms involving NTs in the airway innervation and smooth muscle. The ASM has two tasks: producing NTs (BDNF, NT-4, and NT-3) and interacting with them through their receptors (TrkB, TrkC, and p75NTR). BDNF and NT-4 enhance calcium ion concentration, both directly and through the action of NO, and they strengthen the response of human ASM to acetylcholine (ACh). In addition, NTs modulate cellular growth and proliferation with genomic mechanisms [40].

Different factors allow NT expression in the smooth muscle. For example, prolonged hyperoxia in the neonatal period contributes to airway hyper-responsiveness (characterized by increased contraction and impaired relaxation of ASM) and to neonatal lung injury. In fact, exposure to high concentrations of oxygen increases the BDNF levels and the peribronchial smooth muscle TrkB expression; consequently, the BDNF pathway increases ACh production, enhances the contractility of lung parenchymal strips, and impairs bronchial relaxation via cAMP signaling [41]. Inflammatory cytokines generally promote NT expression in ASM: IL-1β increases NGF and BDNF levels, while TNF-α enhances BDNF and NT-3. These cytokines (especially TNF-α) are now recognized as key players in the pathogenesis of asthma, and together with NTs, they may contribute to molecular alterations in the airway epithelium and smooth muscle. During allergic asthma, the increase in NTs also involves bronchial epithelium and immune cells. Specifically, high levels of NGF and BDNF have been found in the airways of asthmatic patients, in the nasal cavities, and in the subepithelial structures of the skin. This higher specific NT expression, secondary to different biological stimuli, seems to be related to the development of several diseases, such as allergic asthma and chronic rhinitis [42]. Inflammatory processes caused by allergic or viral conditions may also promote NT expression in the neurons of the airway innervation. Immunohistochemical studies of the human lung demonstrated the presence of high levels of NGF and BDNF in peripheral neurons and to a lesser extent in neurons of parasympathetic ganglia [35]. The ganglia express p75NTR receptors, while nerve fibers of the sympathetic nervous system have shown immunoreactivity for both TrkB and p75NTR. These data could underline the different roles of NTs in the nervous system of the airways [43]. Particularly, NGF increases the substantia P (SP) level in vagal afferent fibers, while neurokinin 1 (NK1) receptor expression determines neuromediated airway hyper-responsiveness. SP is a molecular mediator of the non-adrenergic/non-cholinergic (NANC) pathway: NTs increase SP production, which contributes to local inflammation, immunomodulation, and edema, inducing airway hyper-responsiveness and promoting the onset of inflammatory diseases, such as asthma. Furthermore, NTs may increase NK1 receptor expression, and during respiratory inflammatory diseases (such as asthma or bronchitis), may potentiate the growth of airway nerves thanks to the NK receptor signaling pathways. Instead, NT-3 induces neurotransmitter release [13].

NGF and BDNF perform important functions in the neural control of the airways. BDNF may be produced by the nerves of the airways and by the bronchial epithelium. As a growth factor, BDNF may improve the survival or expansion of receptive fields of sensory nerve endings. Furthermore, given that neuronal activity itself can enhance BDNF expression and release, a positive feedback cycle may be set up during inflammatory diseases. BDNF may cause bronchoconstriction by improving receptor expression, increasing synaptic transmission, and inducing cholinergic neurotransmitter release. Both BDNF and NGF promote NK receptor expression [44].

NTs also play a pivotal role in the airway innervation during the development of the lungs in the pre-natal period. A study in mice reported that BDNF-knockout embryos showed a reduction in afferent nerves involved in the control of breathing. Consequently, these embryos developed severe respiratory abnormalities resulting in hypoventilation and irregular breathing. In summary, BDNF and NT-4 with their receptor (TrkB) play an important role in the early development of the lungs, stimulating the differentiation and the maintenance of the sensory and sympathetic neurons that are responsible for respiratory innervation [45]. Immunocytochemical studies demonstrated the expression of BDNF and NT-3 in the intima and adventitia layers of the human pulmonary artery. The presence of NT receptors in these vascular layers suggests a local autocrine or paracrine effect of NTs that could act towards maintaining normal pulmonary vascular tone; this observation may be important for the definition of some vascular diseases, such as pulmonary hypertension. These effects may be explained by the role of NTs in NO production in inducing endothelial cell proliferation and survival, and in controlling vascular tone [46].

Another role of NTs is the regulation of vascular wall structure. In spontaneously hypertensive rats, it was reported that NGF and BDNF are able to increase the pulmonary vasculature and determine a vascular remodeling process, which include pronounced medial thickening. Furthermore, NGF induces angiogenesis, as seen when studying the development of pulmonary vascular components. NTs are involved in pulmonary vasodilation, which can be achieved by endothelial NO production; they also have a role in the contraction and proliferation of vascular smooth muscle. In conclusion, NGF-TrkA and BDNF-TrkB interactions can also upregulate endothelial nitric oxide synthase (eNOS) expression and promote endothelial cell proliferation and survival [46,47].

As mentioned previously, NTs are expressed by a wide number of lung cells, including immune cells, and they seem to be involved in the development of several airway diseases, in both children and adults [13]. A baseline production of NTs has been documented in all types of immune cells, with higher levels after allergic stimulation. Alveolar macrophages express NT-3 in basal conditions, but they also produce NGF and BDNF in response to allergen challenge, while interstitial macrophages constitutively express BDNF [48]. A switch to a Th2 cell response seems to increase NGF secretion, and this observation may be relevant concerning allergic asthma [49]. Mast cells produce NGF, BDNF, and NT-3. When allergens interact with IgE-receptors, a higher level of NGF is detected, suggesting that allergens induce the release of NTs in diseases such as asthma or rhinitis [13]. Allergic asthma and rhinitis are characterized by high NGF and BDNF levels in both serum and bronchoalveolar lavage fluid, especially after allergic stimulation [50,51]. Allergens may induce the release of NGF and BDNF by alveolar macrophages and B-cells, and NGF production by Th2 cells and eosinophils. All NT receptors (i.e., p75NTR, TrkA, TrkB, and TrkC) are expressed on eosinophils; particularly, TrkA receptor expression increases in patients with allergic asthma and rhinitis. NGF induces IL-4 release by eosinophils and promotes, in cooperation with NT-3 and NT-4, eosinophil survival, especially during allergic rhinitis [13]. Thanks to their binding with NTs, eosinophils become the target of various antiapoptotic cellular signals. These events induce the elongation of the half-life of eosinophils and accelerate their recruitment, permitting the beginning and maintenance of the allergic response. High levels of NGF have been found in the blood eosinophils of patients affected by atopic dermatitis, and in this group of patients, the eosinophil–NT interaction may be considered a potential cause of the onset and persistence of itching [52].

### 4.3. NTs and Other Tissues

NTs and their specific receptors are expressed in different tissues (skin, gastrointestinal and urogenital systems, smooth and striated muscle) and cells.

In the gastrointestinal system, NTs control the enteric nervous system (ENS), promoting the differentiation, growth, and survival of peripheral neurons. In Infantile Hypertrophic Pyloric Stenosis (IHPS), the abnormal production of NTs could be responsible for the delay in the structural and functional maturation of the pyloric innervation. In addition, in this condition, the lack of TrkA-positive nerve fibers in pyloric muscle may explain the abnormal intramuscular innervation [52,53,54].

Mast cells are immune cells with the ability to synthetize and release NTs and their receptors. These immune cells are particularly frequent in the skin and in the gastrointestinal mucosa: the skin mast cells can produce TrkA, TrkB, and TrkC, which interact with NGF, NT-4, and NT-3, respectively; the intestinal mast cells synthetize TrkA and TrkC. In some of the pathological conditions involving mast cells, such as mastocytosis, a higher expression of these NTs and their receptors has been described. In mastocytosis, it seems that an increased expression of modified Trk receptors on skin and gut mast cells may contribute to the pathophysiology of this disease [55,56]. In other inflammatory skin conditions, such as atopic dermatitis, NTs and neuropeptide-positive nerve fibers are increased. In atopic dermatitis, NTs modulate the functional role of eosinophils as main target effector cells, and a significant correlation between NGF levels and the eosinophil major basic protein has been described in the eosinophils of affected patients [57,58,59].

### 4.4. NTs and the Brain–Lung Axis

The brainstem respiratory-related nuclei are involved in the modulation of breathing, and they are influenced by different exogenous and endogenous factors. NTs, in particular, BDNF, are crucial for the development and the regulation of these brainstem nuclei from intrauterine life [60]. BDNF seems to increase cholinergic neurotransmission in the brainstem and in other sites of the brain, such as the hippocampus. It may facilitate glutamatergic synaptic transmission via both pre- and postsynaptic mechanisms, but it may attenuate the GABAergic and glycinergic inhibitory transmission [60].

The pontine Koölliker–Fuse nucleus (KFN) is involved in regulating normal breathing. During intrauterine life, the KFN inhibits any respiratory reflex by interfering with central and peripheral chemoreceptors, already fully developed and operative. At birth, the KFN becomes active as a respiratory center that helps in beginning ventilatory activity thanks to connections with the other respiratory-related nuclei [60]. Different literature studies have also shown BDNF action in KF neurons after birth. Lavezzi et al. have reported that BDNF pathway dysfunction may affect the KFN, inducing morphological alterations; however, BDNF is well expressed during all stages of life in other respiratory structures, such as the pre-Boötzinger complex (PBC) [60].

The PBC is a limited region in the ventral–lateral lower brainstem that contains neurons essential for respiratory rhythmogenesis. In the fetal mouse brainstem, BDNF was described as a possible acute regulator of respiratory rhythmogenesis through the potentiation of excitatory glutamatergic synaptic transmission [55,56,60]. The decrease in BDNF expression coincides with a significant reduction in excitatory neurotransmitters (i.e., glutamate) and a sudden and significant increase in inhibitory neurotransmitters (i.e., GABA and glycine receptors) in these same nuclei. The NT downregulation in these nuclei may contribute to the neurochemical, ventilatory, and electrophysiological changes in the respiratory network [60].

## 5. The Role of NTs in Pregnancy

During pregnancy, NTs influence different processes in the preimplantation phase and in the development and maturation of the feto-placental unit. Particularly, BDNF-TrkB high-affinity signaling is responsible for blastocyst outgrowth. TrkB is expressed on trophectoderm cells, and it promotes the suppression of apoptosis in the embryo and the development of the fetus [61,62]. NTs and TrkB are expressed in trophoblast cells and in the placenta during all stages of pregnancy, and they are also involved in fetal growth [61]. As observed in mice, maternal blood BDNF takes part in fetal development by crossing through the feto-placental barrier. Kodomari et al. documented that maternal BDNF can reach the fetal brain in BDNF-knockout mice [63]. Mayeur et al. hypothesized that maternal NTs, which are found in the amniotic fluid, may be transferred to the fetus through two possible mechanisms: through permeable fetal skin or by the fetal ingestion of the amniotic fluid [64].

On evaluating the different stages of pregnancy, BDNF levels are lower in the first trimester than in the second one, and this difference is related to the central role of fetal and placental production [65]. For the same reason, different amounts of NTs have been detected in the human umbilical cord, and lower levels have been found in preterm infants [66,67,68].

### 5.1. NTs and Airway Development

BDNF and NT-4 are also secreted by the trachea and proximal pulmonary lobes, with an important impact on lung development.

Prakash et al. documented BDNF/TrkB complex expression in the embryonic lung from the 12th week of gestation, which is considered to be the period of initial lung development [44]. In BDNF-knockout mice, Radzikinas et al. showed that BDNF is expressed in the lung mesenchyme before its differentiation into smooth muscle fiber (gestation day 11 in mice, and week 5 in humans). Moreover, BDNF expression in the embryonic lung seems to be post-transcriptionally downregulated after ASM differentiation via a mechanism involving microRNA 206 [5,69].

The importance of NTs in lung maturation is demonstrated by several studies on animal models. Homozygous BDNF-knockout mice exhibited multiple structural anomalies such as a thinner bronchial epithelium, a larger air space, a lack of neuroepithelial bodies, and a significant reduction in nerve fiber density in the bronchial smooth muscle, submucous plexus in the bronchioles, and pulmonary artery walls. In addition, a reduction in the subset of afferent nerves involved in lung ventilator control was found, leading to severe respiratory abnormalities, such as irregular breathing and reduced chemosensory drive [5,70].

However, during lung morphogenesis, another impactful role is played by p75NTR, which is essential for lung development [5,42].

### 5.2. NTs and Lung–Brain Axis Development during Pregnancy

The development of the lungs is strictly associated with peripheral neuronal network maturation. The respiratory tract is innervated by two types of neurons: the intrinsic neurons, whose cell bodies are localized along the smooth muscle stripe in the trachea and in the major bronchi, with short axons to innervate nearby targets, and the extrinsic neurons, located in jugular/nodose ganglia, dorsal root ganglia, and sympathetic ganglia, which innervate ASM and neuroendocrine cells in the lungs.

NTs are involved in the embryonic development of neural networks, including the lungs and both the peripheral and the central nervous system. They control the regular maturation of the respiratory network and the primary generation of respiratory rhythm at birth.

During embryogenesis, BDNF and NT-3 are involved in the early stages of brain development together with their high-affinity receptors, TrkB and TrkC, respectively, expressed in the neural plate and in the neural tube. NT-3 is implicated in motoneuron differentiation, while BDNF is responsible for the increase in the number of motoneurons in the neural tube [21]. In the CNS, BDNF supports neuronal differentiation and growth, synaptic and structural plasticity, and higher neuronal functions [22].

NGF is also involved in the development of both the peripheral and central nervous system, playing an important trophic role. This NT is responsible in the peripheral nervous system for the development and the phenotypical maintenance of nociceptive and sympathetic neurons, while in the CNS, NGF is involved in the growth and survival of cholinergic neurons [71].

The PBC is another important component of the respiratory network. In particular, some neurons, which present pacemaker properties, are involved in respiratory rhythm modulation. In the first stage of pregnancy, it has been observed that BDNF plays a fundamental role by enhancing the excitatory glutamatergic synaptic drive and the intrinsic bursting frequency of isolated endogenous bursting neurons. This causes an increase in the respiratory frequency in contrast to what happens in the postnatal ages. Furthermore, the PBC is a site of the production of BDNF [59].

BDNF action has also been documented in the KFN, which is well developed at the 25th week of gestation. During pregnancy, the KFN suppresses any respiratory reflex by inhibiting central and peripheral chemoreceptors. BNDF is essential for KFN maturation in intrauterine life, but it is not involved in the onset of the breathing process promoted by the KFN in the postnatal period. In fact, a high expression of BDNF has been found in utero, while its expression is lower in newborns [60].

A5 noradrenergic neurons are a group of cells localized in the ventrolateral pons, and are involved in cardiorespiratory control. These neurons are responsible for the modulation of respiratory rhythmogenesis through the inhibition of the medullary respiratory pattern generator. Lesions in this area are associated with an increased respiratory frequency [72].

Other NTs are involved in lung development in the fetus. Glial cell line-derived neurotrophic factor (GDNF) is expressed in both the central and peripheral nervous system. Glazner et al. have documented the presence of GDNF mRNA in the motoneurons of the spinal cord and brainstem nuclei that are involved in respiratory skeletal muscle innervation [73]. According to Maxwell et al., GDNF takes part in fetal development by promoting the noradrenergic differentiation of A5 noradrenergic neurons [74]. Moreover, Huang et al. showed that GDNF genetic loss decreases the number of A5 noradrenergic neurons in the pontine nucleus. However, GDNF requires BDNF expression to act as a cofactor in regulating the phenotypic differentiation of A5 noradrenergic neurons [72].

## 6. NTs in the Neonatal Period

Prematurity is a condition characterized by a delicate balance between neuroprotection and organ damage [75,76,77]. BDNF and NT-3 seem to have a crucial role in neuroprotection by promoting neuronal survival and by reducing neuronal apoptosis. BDNF levels are higher in the third trimester of pregnancy and in neonates whose mothers received a complete course of antenatal steroids; conversely, low BDNF levels were associated with both chorioamnionitis and mild intraventricular hemorrhage (IVH) [65]. Moreover, NT-3 levels seem to be higher in term infants [64]. Therefore, both BDNF and NT-3 may be considered possible markers of neuronal maturation.

The correlation between the NT levels and neonatal lung maturation is, to date, open to debate. As animal models have shown, BDNF modulates glutamatergic neurotransmission in the KFN, which has an essential role in the respiratory network. Kron et al. described the progressive desensitization of NMDA currents in the presence of BDNF after the first 2 days of life [75]. In another study, Kron et al. demonstrated changes in inhibitory postsynaptic currents (IPSCs) in the KFN during the first days of life. In the postnatal period, BDNF reduces the spontaneous IPSC frequency, while the evoked IPSCs show developmental differences; later on, the response pattern becomes heterogeneous [78]. The PBC in the caudal ventrolateral medulla seems to be influenced by BDNF levels. Thoby-Brisson et al. demonstrated that the respiratory rhythm generated in vitro was increased by 30% after BDNF administration, but not in the presence of TrkB [79]. In rat models, BDNF and its high-affinity receptor TrkB are increased in the first postnatal week, but lowered later on [50]. Furthermore, BDNF and TrkB could be involved in brain–lung axis development at birth. However, after the first week of life, a decrease in BDNF and TrkB expression was described, especially in the PBC; nucleus; ambiguous, commissural, and ventrolateral subnuclei of the solitary tract nucleus; and retrotrapezoid nucleus. These data may partially explain the inhibitory–excitatory imbalance in the respiratory network during the first period of life [53]. The expression of BDNF and TrkB receptors is also documented in airway vagal preganglionic neurons (AVPNs), suggesting their potential role in cholinergic outflow regulation [80]. In addition, in vitro models have shown an influence on calcium influx when airway smooth cells are exposed to BDNF [40].

### 6.1. NTs and Neonatal Lung Diseases

The discovery of the brain–lung axis has allowed us to better define the pathophysiology of some important neonatal lung diseases.

Bronchopulmonary dysplasia (BPD) is a chronic lung disease which affects newborns and is caused by lung damage related to high oxygen levels [81,82]. The major repercussions of BPD in neonatal life are well known, but the exact pathological mechanism at the origin of this disease is not fully understood. A strong association between BPD and asthma in adults was described [82,83], but the way hyperoxia induces the damage is still being debated. Studies on rat pups exposed to high oxygen levels have shown that the cholinergic regulation of airway contractile responses is upregulated after exposure to hyperoxia [84,85]. The role of NTs in neonatal lung damage has been described by many authors. Exposure to hyperoxia in rat pups has been found to induce a significant alteration in neurochemical pathways in brainstem respiratory-related nuclei, but there has been no documented modification of non-respiratory ones. A significant difference in BDNF immunoreactivity was found in non-respiratory cuneate nuclei, showing an important difference between rats exposed to high oxygen levels and rats which were not. In fact, in the first group, BDNF decreased 2 days later than in the second one [84,85]. Hyperoxia also increases BDNF and TrkB receptor expression in ASMs, but there is no documented modification in NGF levels [84,85]. The reversal of ASM contraction in rats exposed to hyperoxia and K252a, a tyrosine kinase inhibitor able to block BDNF-TrkB signaling, may partially explain the contractile response of lung parenchyma in neonatal lung injury [86].

**Figure 1:**
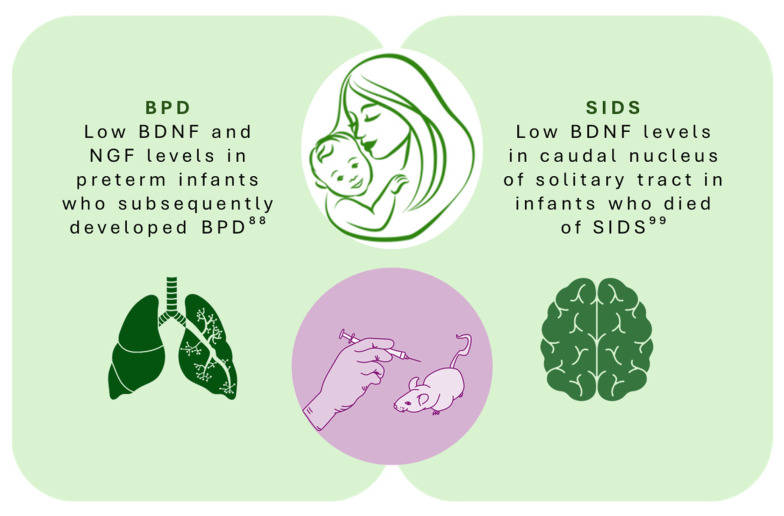
NT and newborns diseases.

Human studies also supported the correlation between the concentration of some NTs at birth and the development of BPD. D’Angio et al. evaluated the plasma cytokine profiles from 943 neonates born at ≤1000 g and surviving to 28 days. The median BDNF levels were consistently below the IQR in newborns with no lung disease, while they were doubled in the classic BPD group. Altered levels of IL-6, IL-8, IL-10, and RANTES were identified among infants who subsequently developed BPD [87]. In another study, BDNF and NGF concentrations at birth differed significantly between groups: BPD preterm infants had lower BDNF levels than preterm infants without BPD or term infants (Figure 1). High-BDNF levels were associated with a low probability of ventilation, and high NGF levels were linked to fewer days of ventilation. A positive correlation between NGF concentration at birth, the Bayley Scale of Infant Development—Third Edition [88] (used to assess developmental infant functioning), and language composite scores at 24 months was also described [89].

The results of these studies allow us to hypothesize anout the neuroprotective role of drugs with lung action in patients at risk of BPD. Inhaled nitric oxide (iNO) seems to promote myelination and neuroprotection in neonatal animal models [90]. Apparently, iNO influences the early upregulation of NTs, helping to maintain BDNF levels, as described for rat pups not exposed to hyperoxia [91,92]. In contrast with its potential positive role on neuronal development, preterm infants who have been treated with dexamethasone seem to develop impairments in neuromotor and cognitive skills at school age. In fact, preterm infants have lower NT levels that could be further suppressed by dexamethasone treatment in order to prevent BPD [93].

### 6.2. NT and Other Newborns Diseases

Sudden infant death syndrome (SIDS) is the sudden and unexpected death of a healthy child under one year of age, usually during sleep. The cause of SIDS is still unknown, but many risk factors have been identified, including prematurity [93,94,95,96,97].

In animal models, it has been observed that NTs may play a role in causing SIDS through various mechanisms. Studies in mice have shown how nicotine exposure during pregnancy promotes the occurrence of central apnea, which can lead to lethal respiratory arrest under hypoxic conditions. This occurs through the increased synthesis of bronchopulmonary C-fibers (PCFs) and transient receptor potential vanilloid 1 (TRPV1) expression via the upregulation of neural TrkA and downregulation of pulmonary BDNF and NGF [98]. A reduction in cigarette smoke exposure during pregnancy may thus reduce brain alterations in the offspring. In a group of rat pups not subjected to hyperoxia compared to those subjected to hyperoxia at birth, levels of cytochrome oxidase, BDNF, TrkB, and several serotonergic proteins (5-HT1A and 2A receptors, tryptophan hydroxylase [TPH]-synthesizing enzyme, and serotonin transporter [SERT]) decreased in various brainstem nuclei involved in the breathing mechanism during the critical postnatal period. Then, neonatal hyperoxia reduces neither the risk of respiratory apnea nor the alterations in different brainstem nuclei involved in the genesis of the respiratory impulse [86].

The involvement of some NTs has also been hypothesized in post-mortem research involving infants affected by SIDS. Significant alterations in BDNF levels in the pontine KFN have been reported in a wide number of sudden intrauterine unexplained death syndrome (SIUDS) and SIDS cases [99]. In another study, low BDNF levels in different cerebral regions involved in respiratory control were found in children who died of SIDS. In this latter study, a correlation between maternal nicotine exposure during pregnancy and lower BDNF levels was described [100,101]. Tang et al. evaluated the concentrations of BDNF and TrkB in a group of children who died of SIDS compared to non-SIDS children, and they noted lower BDNF values in the caudal nucleus of the solitary tract and higher TrkB expression in the caudal dorsal motor nucleus of the vagus in SIDS infants (Figure 1). These results reinforce the possibility that an abnormal concentration of BDNF and TrkB may be an expression of reduced neuroprotection in breathing centers in infants with SIDS [100] and could support the use of BDNF levels to identify patients with risk factors for SIDS in future studies.

Congenital central hypoventilation syndrome (CCHS), also known as Ondine’s syndrome, is a rare disease resulting from alveolar hypoventilation due to a congenital deficit in the central autonomic control of ventilation and global autonomic dysfunction [102]. BDNF seems to be implicated in the control of neuronal plasticity after hypoxia, and several authors have found a reduction in BDNF expression in the cerebrospinal fluid of patients affected by CCHS compared to healthy groups [103,104].

## 7. Conclusions

Since their first description in the 1950s by Rita Levi-Montalcini and Viktor Hamburger [105], NTs have been investigated by many studies, with the purpose of defining their physiological and pathological implications in different human conditions. The evidence summarized in this article confirms the involvement of NTs in the lung–brain axis, and their crucial role in its dysfunction. Further studies are needed to better define the application of NTs as useful biomarkers in anticipating and defining important infant pathologies.

## Figures and Tables

**Table 1:** Interactions between NTs and their receptors [5,10,11].

Receptor	NTs	Mechanism of Action	Effects
p75NTR	All NTs and pro-NTs with low affinity	NF-kB pathway, Jun Kinase pathway	Cell growth (NF-kB), cell apoptosis (Jun Kinase)
TrkA	Preferentially NGF	RAS/MAP/ERK pathway, PI3K pathway	Cell differentiation and replication, apoptosis
TrkB	Preferentially BDNF and NT-4		
TrkC	Preferentially NT-3		

p75NTR = p75 neurotrophin receptor; TrkA = Tropomyosin receptor kinase A; TrkB = Tropomyosin receptor kinase B; TrkC = Tropomyosin receptor kinase C; NTs = neurotrophins; NGF = nerve growth factor; BDNF = brain-derived neurotrophic factor; NT-3 = neurotrophin-3; NT-4 = neurotrophin-4; PI3K = phosphoinositide 3-kinase; NF-kB = Nuclear Factor kappa B.

**Table 2:** Roles of NTs in the different lung cells [5,35].

Lung Cells	Roles of NTs
Airway epithelium	Lung development Regulation of epithelial cell proliferation or survival Clara cell proliferation Repair of epithelial cell injury Nitric oxide production Airway remodeling
Airway smooth muscle	Airway development and remodeling Bronchoconstriction Cell proliferation
Airway innervation	Neurokinin 1 receptor expression Increasing Substantia P in vagal afferents Cholinergic neurotransmitter release Improvement of survival or expansion of receptive fields of sensory nerve endings Support of the differentiation and maintenance of sensory and sympathetic neurons during embryological development
Endothelium	Control of pulmonary vascular tone Upregulation of eNOS expression and vasodilatation Contraction of vascular smooth muscle Promotion of endothelial cell proliferation and survival
Immunological cells	Induction of interleukin release from eosinophils Promotion of eosinophil survival Promotion of the response of antiapoptotic cellular signals Improvement of NT production after allergenic stimulations

eNOS = endothelial nitric oxide synthase; NTs = neurotrophins.
